# Quality of Life, Physical Diseases, and Psychological Impairment among Survivors 3 Years after Wenchuan Earthquake: A Population Based Survey

**DOI:** 10.1371/journal.pone.0043081

**Published:** 2012-08-21

**Authors:** Jin Wen, Ying-kang Shi, You-ping Li, Ping Yuan, Fang Wang

**Affiliations:** 1 Department of Hospital Management and Health Policy, West China Hospital, Sichuan University, Chengdu, People's Republic of China; 2 The Chinese Evidence-Based Medicine Center and Department of Clinical Epidemiology, West China Hospital, Sichuan University, Chengdu, People's Republic of China; 3 Department of Epidemiology and Biostatistics, West China School of Public Health, Sichuan University, Chengdu, People's Republic of China; Universidad Europea de Madrid, Spain

## Abstract

**Background:**

Few research studies have addressed the long-term effects caused by catastrophes, and no study has ever explored the life quality, physical diseases, and psychological impairment of earthquake survivors at the same time. This study seeks to reveal survivors' quality of life, physical diseases, and mental health.

**Methods:**

A cross-sectional survey was conducted through multi-stage sampling approach three years after the Wenchuan earthquake.

**Results:**

A total of 2525 subjects were interviewed. Symptoms of PTSD were reported by 8.8% of the respondents from the seriously affected areas and 0.5%, the less hit areas. Prevalence of chronic diseases was 39.2% and 22.1% respectively, and two-week prevalence rate, 24.9% and 12.7% respectively. In the multivariate analysis, two-week prevalence, displacement, no regular income, receiving mental health support after the disaster, family members died or missing, injured due to the quake, and person who witnessed someone being killed or injured were independently associated with higher prevalence for symptoms of PTSD. Most subscales of SF-12 negatively correlated with age, chronic diseases, two-week prevalence, injured due to the disaster, home or property loss, and score of the 3-year PTSD symptoms, but positively correlated with higher education and higher household income.

**Conclusions:**

The rates of physical diseases and symptoms of PTSD were relatively high, and the quality of life was poor among victims in the hard-hit areas 3 years after the earthquake. Physical impairment correlated with symptom of PTSD, and both were negatively associated with quality of life.

## Introduction

The massive earthquake measuring 8.0 on the Richter scale that hit Wenchuan on 12 May 2008 had affected 417 counties and districts in ten provinces, covering some 500 000 km^2^ and left millions of people homeless. As of 25 August 2008, 69 226 people were killed, 374 643 injured and 17 923 missing.

Shortly after the disaster, Chinese government allocated more than 300 billion Yuan from the central budget for the reconstruction of the most seriously affected counties and assembled a special working group. Meanwhile, 19 provinces and municipalities partnered with the earthquake affected areas in the course of reconstruction. They've managed to rebuild 1.9 million houses in villages, and more than 200 thousand in cities within three years after the catastrophe. Over two thousand hospitals have also been rebuilt [1]. As a result, the accessibility and availability of medical services in the earthquake-stricken areas have improved significantly. Moreover, health insurance has covered the majority (94%) of the population [2].

While the Chinese people have achieved a reconstruction miracle, local survivors' long-term health status, including physical disease, mental health, and quality of life, are to be revealed. A recent systematic review [3] showed that the number of health related academic articles dropped dramatically 2 years after the earthquake, which, to some extent, reflected, that researchers ignored the long term impact of a disaster to the affected population. Previous researches have shown that post traumatic stress disorder (PTSD) and other psychological health problems were common among earthquake survivors [4]–[8], and some of them demonstrated that quality of life tended to be worsen or influenced by the mental impairment [9], [10]. Nevertheless, up to now, to the best of our knowledge, no researches have addressed earthquake survivors' quality of life, physical diseases, and psychological impairment simultaneously.

China tops the list of the number of devastating earthquakes, but the number of researches on long-term disaster related health problems was very few. A comprehensive understanding of survivors' health status is essential for identifying vulnerable populations and developing culturally specific health interventions. As a public health response, a population-based survey was conducted to reveal the prevalence of symptoms of PTSD, physical disease, quality of life, and associated factors among random samples in the seriously hard-hit areas and less affected ones.

## Methods

### Ethics Statement

This study protocol was approved as a less than minimal risk research by the Institutional Review Board (IRB) of West China Hospital in Sichuan University. Many of the people in rural areas of west China and the earthquake-affected areas are illiterate. Written consent is not common practice and may violate confidentiality. Therefore, a consent form to obtain verbal consent from respondents was proposed and approved by the IRB, together with the study protocol. Prior to the interview, each investigator read carefully the consent form, which contains information on the objectives of the study, the selection process, risks, benefits and freedom of the participation, as well as information on confidentiality.

### Study design and participants

**Figure 1:**
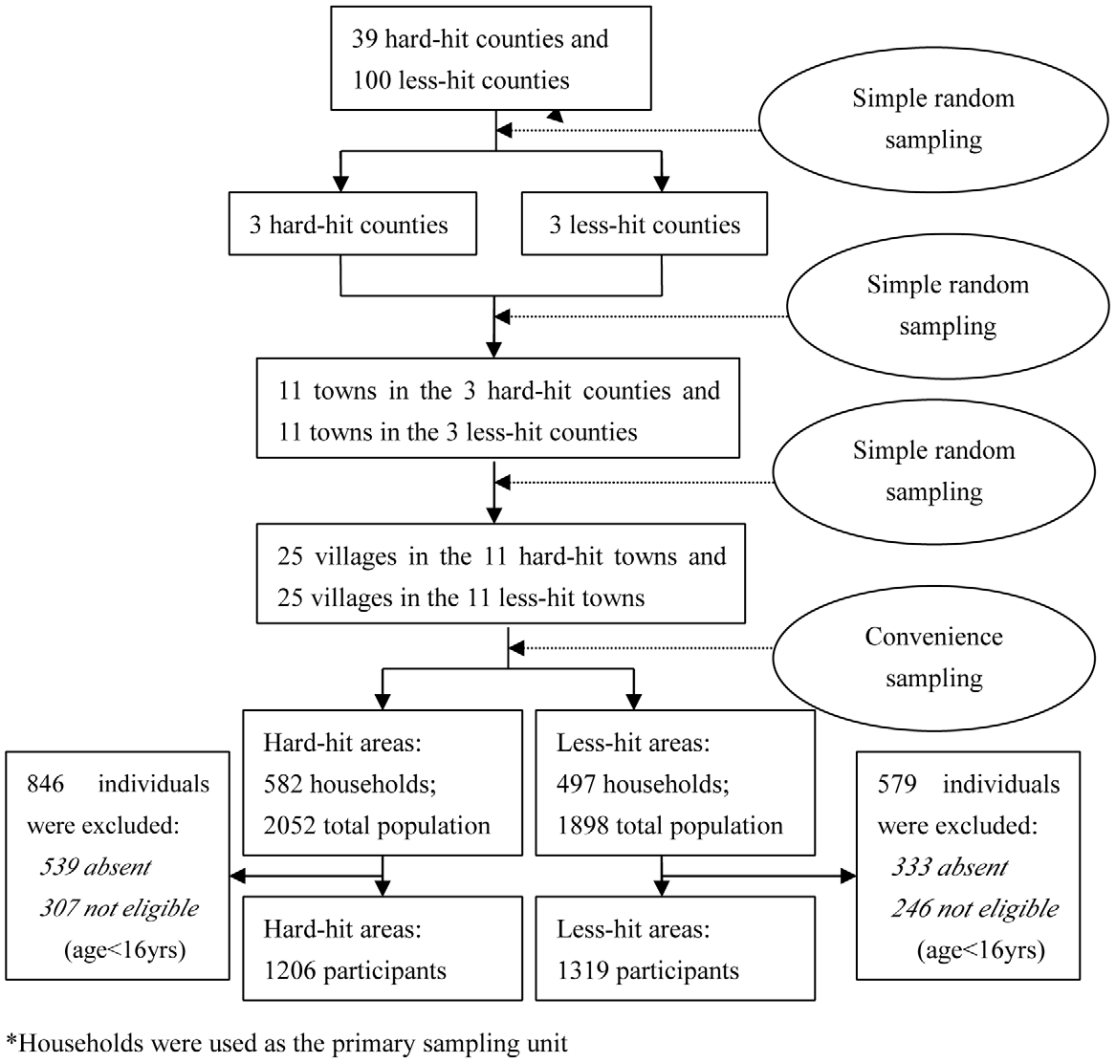
Flow chart of sampling stages. *Households were used as the primary sampling unit.

A cross-sectional survey was conducted from May to June 2011, three years after the disaster. The study recruited participants based on households in the earthquake stricken rural areas, including the seriously hard-hit counties (Wenchuan, Shifang, and Mianzhu) and the less-hit counties (Shuangliu, Xindu, and Qingshen). The survey was designed and conducted by a multidisciplinary group that consisted of epidemiologists, psychologists, biostatisticians, sociologists, and physicians. All data were collected through face-to-face interview questionnaires, and all interviewers were trained before the pilot study and formal study. Subjects must have been present at the time of the earthquake and above the age of 7 at the time of interview. Subject was ineligible if he/she was diagnosed mental health impairment before the disaster. A multi-stage cluster sampling approach was adopted (figure 1). As most earthquake affected regions are located in high mountains or deep valleys with slope of at least 60°, it was impossible to take a random sample in the final stage, during which we selected households based on convenience and consultation with local healthcare providers to assure sample's representativeness.

### Measures

According to our definition, chronic diseases should fall into the following list: chronic non-specific lung disease (asthma, bronchitis and pulmonary emphysema), cardiac diseases, atherosclerotic disease, cerebrovascular disease (stroke, excluding transient ischemic attacks), diabetes mellitus, malignant neoplasms, osteoarthritis, and rheumatoid arthritis. All the above chronic diseases should be diagnosed by physicians in hospitals or clinics before the investigation with current symptoms or treatments. Two-week morbidity was investigated by asking the interviewees whether members of their households had been ill in the previous two weeks, and measured based on the number of people ill in the past two weeks.

We adopted the instrument of PTSD Checklist-Civilian Version (PCL-C) to measure the symptoms of PTSD [11]. The instrument is a self-report 17-item symptom scale that corresponds to DSM-IV criteria [12], and is often used when a clinical interview is not feasible [13],[14]. Total score ranges from 17 to 85, and an adult with a score of 38 or greater was classified as having probable PTSD [13], [15]. The Internal consistency (Cronbach's Alpha coefficient) of the PCL-C in our study was 0.80.

The short form 12-v2 (SF12-v2) was used to measure the quality of life (QOL), which was a shorter form with 12 questions all selected from SF-36 [16]. It provided a glimpse into the mental and physical functioning and overall health-related-quality of life. The 12 questions were combined, scored, and weighted to create eight subscales (general health perceptions (GH), physical functioning (PF), role limitations due to physical problems (RP), bodily pain (BP), vitality (VT), social functioning (SF), role limitations due to emotional problems (RE) and mental health (MH)) and two components (Physical Component Summary (PCS) and Mental Component Summary (MCS)). The two summary components of PCS and MCS and the eight subscales, including PF, RP, BP, GH, VT, SF, RE and MH, were scored based on the US norm [17], with higher scores indicating better QOL.

To address potential sources of bias and ensure the quality of this study, a number of approaches were used. At the stage of developing questionnaire, we organized an inter-disciplinary group to make the items understandable, representative, and objective. Before the formal investigation, all the interviewers were trained systematically and a pilot study was performed. When conducting field interview, a local healthcare provider who was quite familiar with the local residents was invited to explain the purpose of this study to participants. This contributed to a high response rate (91.3%) in total. We also adopted a multistage sampling method to reduce selection bias and multivariate analyses to minimize the effects of confounders.

### Sample size

The sample size was calculated on the basis of an assumed PTSD prevalence of 10% in the hard-stricken areas and 6% in the less-hit areas, a value of 0.9 for power and 0.05 for alpha (significance level) as well. The initially estimated sample size was 1,014 for each group. However, we increased the primary sample size by 15% to avoid the loss of potential non-respondents. Thus our minimum sample size for each group was 1,166 subjects. Estimates of PTSD prevalence in earthquake hard hit areas within a short term after the disaster varied from 9.4% to 45.5% [18], [19]. Considering the potential time effect, we used 10% prevalence in hard-hit area and 6% in less-stricken area respectively to calculate the sample size of our survey. Households were used as the primary sampling units.

### Statistical analysis

Frequencies, percentages, means and standard deviations were calculated for descriptive data, t-tests were used to evaluate differences in continuous variables, and Chi-square tests were used to test for significance in categorical data. If continuous variables were not normally distributed, Mann-Whitney U tests were applied. Backward stepwise multivariate logistic regression and multiple linear regression were used to identify risk factors for PTSD symptoms and quality of life, respectively. P<0.05 was considered to be statistically significant. Data were analyzed with SPSS version 17.0 (SPSS Inc, Chicago, Ill).

## Results

Of the 1206 eligible respondents in hard-hit area (response rate, 93.8%), 44.9% were male, 58.8% received primary or lower level of education, and 17.2% were minorities; their mean age (SD) was 46.4(16.5) (range, 16–90). Of the 1319 respondents in less-hit area (response rate, 89.1%), 48.7% were male, 52.2% received primary or lower level of education, and 1.6% were minorities; their mean age (SD) was 47.9(16.8) (range, 16–94) (table 1). In almost all cases, absence or age under 16 at the time of the interview was the reason for nonparticipation. The mean age and sex of hard-hit and less-hit area participants were not significantly different from the nonparticipants.

**Table 1:** Demographic characteristics of the populations of hart-hit and less-hit areas, Sichuan Province, China 3 years after Wenchuan earthquake.

Characteristic	Hard-hit area	Less-hit area	p-value
Gender			0.056
Male, No. (%)	542(44.9)	643(48.7)	
Female, No. (%)	664(55.1)	676(51.3)	
Age, mean (SD), y	46.4(16.5)	47.9(16.8)	0.032
Education			0.001
Primary school or lower, No. (%)	709(58.8)	689(52.2)	
Higher than primary school, No. (%)	497(41.2)	630(47.8)	
Ethnicity			<0.001
Han, No. (%)	999(82.8)	1298(98.4)	
Minorities, No. (%)	207(17.2)	21(1.6)	
Household income			<0.001
RMB0–5000, No. (%)	156(12.9)	142(10.8)	
RMB5000–20000, No. (%)	746(61.9)	564(42.8)	
RMB20000–50000, No. (%)	266(22.1)	540(40.9)	
RMB≥50000, No. (%)	38(3.2)	73(5.5)	

Symptoms of PTSD were reported by 8.8% of the participants in the hard affected areas and 0.5% of those in less hit areas, respectively. The subscale and total scores of PTSD symptom are significantly higher in subjects from hard hit areas than those from the less hit areas (all P<0.001). Significant differences in subscales of quality of life between the two groups were demonstrated, except for social functioning; In general, participants in hard hit areas scored lower than those in less hit areas. Meanwhile, prevalence of chronic diseases and two-week prevalence rate were found much higher in severely hit regions than less affected regions (prevalence of chronic disease: 39.2% vs. 22.1; two-week prevalence rate: 24.9% vs. 12.7%). (table 2)

**Table 2:** Descriptive statistics of PTSD, quality of life, and physical diseases among participants from hard-hit and less-hit areas.

Variable	Hard-hit area	Less-hit area	p-value
PTSD symptom			
Re-experiencing, mean (SD)	9.1(3.4)	5.9(1.8)	<0.001
Avoiding/numb, mean (SD)	9.0(2.9)	7.6(1.9)	<0.001
Hyper-arousal, mean (SD)	7.9(3.0)	5.5(1.4)	<0.001
Total score, mean (SD)	26.0(7.8)	19.1(4.5)	<0.001
Quality of life			
Physical functioning, mean (SD)	51.3(9.7)	53.3(8.0)	<0.001
Role physical, mean (SD)	50.5(11.2)	53.8(8.0)	<0.001
Bodily pain, mean (SD)	50.8(10.9)	52.6(7.7)	<0.001
General health, mean (SD)	38.5(12.5)	42.8(11.7)	<0.001
Vitality, mean (SD)	54.7(7.0)	57.1(6.5)	<0.001
Social functioning, mean (SD)	54.5(6.3)	54.0(8.7)	0.119
Role emotional, mean (SD)	45.3(9.6)	48.9(7.9)	<0.001
Mental health, mean (SD)	49.6(7.8)	51.8(7.0)	<0.001
Mental component summary, mean (SD)	49.8(8.2)	51.8(7.7)	<0.001
Physical component summary, mean (SD)	49.3(12.2)	51.8(9.2)	<0.001
Physical diseases			
Chronic diseases, No. (%)	473(39.2)	292(22.1)	<0.001
Two-week prevalence rate, No. (%)	300(24.9)	168(12.7)	<0.001

The results of bivariate analysis for risk factors of PTSD symptoms are shown in table 3. Symptoms of PTSD were significantly higher among those with lower education, lower family income, chronic diseases or illness within two weeks; those who had no regular income after the disaster, received mental health support after the earthquake, lost their family members, homes, properties or witnessed someone being killed or injured in the disaster; as well as females, the displaced, and the injured.

**Table 3:** Risk factors of PTSD symptoms by logistic regression.

	PTSD No.(%)	Bivariate OR (95% CI)	P Value	Multivariate OR(95% CI)	P Value
Gender			0.002	–	
male	37(3.1)	0.53(0.36–0.80)			
female	76(5.7)	1.00			
Age (years)			0.935	–	
<60 yrs	84(4.5)	0.98(0.64–1.51)			
≥60 yrs	29(4.5)	1.00			
Education			0.028	–	
Primary school or lower	74(5.3)	1.56(1.05–2.32)			
Higher than primary school	39(3.5)	1.00			
Ethnicity			0.285		0.015
Han	106(4.6)	1.53(0.70–3.32)		2.70(1.21–6.02)	
Minorities	7(3.1)	1.00		1.00	
Household income			0.001	–	
RMB0–5000	20(6.7)	2.80(1.51–5.17)			
RMB5000–20000	70(5.3)	2.19(1.36–3.54)			
RMB≥20000	23(2.5)	1.00			
Chronic diseases*			0.001	–	
Yes	51(6.7)	1.96(1.34–2.86)			
No	62(3.5)	1.00			
Two-week prevalence rate			<0.001		<0.001
Yes	46(9.8)	3.24(2.19–4.78)		2.45(1.62–3.72)	
No	67(3.3)	1.00		1.00	
Displaced after earthquake			<0.001		0.024
Yes	36(11.3)	3.52(2.32–5.33)		1.69(1.07–2.66)	
No	77(3.5)	1.00		1.00	
Have regular income after earthquake			0.003		0.001
Yes	23(2.7)	0.49(0.31–0.78)		0.45(0.27–0.73)	
No	90(5.4)	1.00		1.00	
Received mental health support after earthquake			<0.001		0.018
Yes	37(13.9)	4.74(3.12–7.19)		1.77(1.10–2.85)	
No	74(3.3)	1.00		1.00	
Family member died or missing during earthquake			<0.001		0.001
Yes	58(11.0)	4.34(2.96–6.37)		2.14(1.37–3.35)	
No	55(2.8)	1.00		1.00	
Injured due to the disaster			<0.001		0.006
Yes	59(10.5)	4.16(2.83–6.09)		1.86(1.19–2.90)	
No	54(2.8)	1.00		1.00	
Lost home or property			<0.001		
Yes	111(5.3)	12.49(3.07–50.74)		–	
No	2(0.4)	1.00			
Witnessed someone being killed or injured			<0.001		0.013
Yes	83(8.6)	4.77(3.12–7.30)		1.96(1.15–3.35)	
No	30(1.9)	1.00		1.00	

* PTSD symptoms: PCL-C score of 17 items ≥38.

Abbreviations: CI, confidence interval; OR, odds ratio; PTSD, posttraumatic stress disorder.

–Variable was not retained in backward stepwise regression procedure.

In the multivariate analysis, two-week prevalence, displacement after the disaster, having no regular income after the quake, receiving mental health support after the disaster, family members died or missing, person who injured due to the quake, and respondents who witnessed someone being killed or injured during the disaster were independently associated with higher prevalence for symptoms of PTSD (table 3).

Table 4 presents the results of multiple-regression analyses to predict the influence of factors on the scores of the QOL subscales and domains 3 years after the earthquake. The results revealed that most subscales negatively correlated with age, chronic diseases, two-week prevalence, injury due to the disaster, home or property loss, and score of the 3-year PTSD symptoms, but positively correlated with higher education and higher household income. The PCS scores negatively correlated with age, chronic diseases, two-week prevalence, injury due to the disaster, and score of the 3-year PTSD symptoms, but positively associated with higher education and household income. Meanwhile, the MCS score negatively correlated with score of the 3-year PTSD symptoms and positively correlated with age.

**Table 4:** Multiple linear regression analysis to predict scores of the subscales and domains of the SF-12 three years after the earthquake.

	PF	RP	BP	GH	VT	SF	RE	MH	PCS	MCS
Age (yrs)	−0.15**	−0.08**	−0.05**	−0.12**	−0.02*	–	0.08**	–	−0.15**	0.09**
Gender (F = 1/M = 0)	–	–	–	–	–	0.52	–	–	–	–
Education (rank from low to high = 1–5)	–	0.61**	0.35	0.79**	0.57**	–	–	–	0.62**	–
Ethnicity (Han = 1/Minorities = 0)	–	–	–	–	–	−0.93	–	−1.21*	–	−1.02
Household income(rank from low to high = 1–3)	0.87**	1.28**	0.77**	–	−0.42*	1.02**	0.93**	0.55*	0.75**	–
Chronic diseases(yes = 1/no = 0)	−4.44**	−6.87**	−4.68**	−9.39**	−1.96**	−1.10**	−1.44**	−1.18**	−7.63**	–
Two-week morbidity (yes = 1/no = 0)	−2.42**	−4.81**	−5.97**	−3.46**	−2.50**	−1.76**	–	−1.02*	−5.11**	–
Displaced after earthquake (yes = 1/no = 0)	–	–	–	−1.25*	−1.11**	–	–	–	–	–
Have regular income after earthquake(yes = 1/no = 0)	–	–	–	1.19**	–	–	–	–	–	–
Received mental health support after tsunami(yes = 1/no = 0)	1.15*	–	–	1.57*	–	–	–	–	1.16	–
Family member died or missing (yes = 1/no = 0)	–	1.14**	1.33**	1.67**	1.06**	–	–	–	1.26**	–
Injured due to the disaster (yes = 1/no = 0)	−3.71**	−3.03**	−2.73**	–	−1.95**	−1.58*	−2.62**	−1.19	−3.14**	−1.30
Lost home or property(yes = 1/no = 0)	−1.12*	–	−2.35**	−3.56**	−1.82**	2.65**	−0.94*	–	−2.25**	–
Witnessed someone being killed or injured(yes = 1/no = 0)	0.72	–	1.93**	–	–	0.92*	–	−0.68*	1.09*	–
PTSD score	−0.13**	−0.22**	−0.24**	−0.27**	−0.15**	−0.11**	−0.41**	−0.18**	−0.17**	−0.26**
Constant	62.70	59.52	60.57	57.36	63.67	52.80	51.92	55.51	62.72	53.66

* p<0.05;

** p<0.01;

– no significant.

## Discussion

This population-based survey revealed higher prevalence of symptoms of PTSD, chronic diseases, and two-week morbidity, and lower quality of life among people in hard-hit areas than those in less affected areas.

Most hard-hit regions of the Wenchuan earthquake are located in remote and rural areas. Because of underdeveloped economy and lack of medical resources, along with the catastrophe, people there were more likely to be attacked by acute and chronic diseases, which contributed to the high prevalence of chronic diseases and two-week morbidity rate. However, Chinese government has made great efforts to reduce the negative impact of the disaster and promote public health. A good example is that the two-week morbidity rate was 27.6% and 21.8% in hard-hit areas and less-hit areas respectively three months after the earthquake [20], higher than the rates three years later revealed by our study.

A significant finding is that the receipt of mental health support after the earthquake was associated with symptoms of PTSD, which is similar to the finding of the tsunami survey [21]. The association was significant even in multivariate analysis and when considering the timing of support. One possible factor might be responsible for this result: lots of volunteers provided mental health consultation and psychological support for earthquake survivors, but not all the volunteers had the professional skills to deliver the service. However, no strong evidence revealed whether providing of mental health support could reduce the prevalence of PTSD, which should definitely be explored in the future.

Bivariate logistic regression revealed that chronic diseases, and multivariate analysis showed that two-week morbidity were associated with symptoms of PTSD. It indicated that the physical diseases might be important risk factors of mental health. In multivariate analysis, displacement was an independent risk factor for symptoms of PTSD. Since Chinese government has provided permanent housing for earthquake victims (especially the displaced persons), this result reminded us that the displaced survivors might be fragile and need to be given more concerns. Meanwhile, this study demonstrated that having regular income after the disaster could significantly reduce the risk of developing PTSD.

The loss of a family member, being injured due to the disaster, and witnessing someone being killed or injured by the earthquake were contributory factors for PTSD symptoms, similar to the findings of a previous study which was conducted 15 months after the Wenchuan earthquake [22].

In our study, the prevalence of 8.8% for symptoms of PTSD among hard-hit areas 3 years after the disaster was lower than that found one year after the earthquake in similar regions [18], [23], which proved that though symptoms of PTSD among survivors were serious, they might decrease as the time goes by.

In the quality of life subscales, prominent potential risk factors were noted, including age, education level, household income, chronic diseases, two-week morbidity, family member dead or missing due to the disaster, injured, prominent financial or home loss immediately after earthquake, and PTSD score. When subjects were elderly, there was a significant negative correlation in the PCS dimension of quality of life but positive correlation in the MCS dimension. Physical illness, including chronic diseases, two-week morbidity, and injured due to earthquake, was a statistically negative association with most of the subscales of quality of life. Notably, a higher PTSD score might be a true risk factor to all subscales of quality of life. A study [24] has shown that financial loss affects physical and psychological health. However, this study demonstrated that financial or property loss only negatively associated with PCS by controlling potential confounders.

Some possible limitations of our study need to be mentioned. Firstly, the instrument of SF-12 was developed and validated in the Western world and cultural factors may therefore play a role in the underreporting or overreporting for symptoms of PSTD. However, this study showed that the Cronbach's Alpha Coefficient for the SF-12 was 0.802, which exceeded the recommended level of 0.70 [25] and was consistent with LUO Xuemei's reports [26], indicating the good internal consistency reliability of the SF-12 in earthquake survivors. Secondly, there was no baseline data of the participants interviewed. As a result, we failed to explore the changes of physical diseases, mental health, and quality of life among survivors directly. Nevertheless, several studies pertaining to the Wenchuan earthquake have reported some useful information on survivors' physical diseases, mental health, or quality of life within a short time after the disaster, providing good comparisons to our findings [18], [20], [23], [27]. An additional drawback is that in this paper we failed to address other mental health problems such as anxiety and depression, though such problems were not our research priorities.

We believe that the strengths of this study are as follows. First, this is a population-based survey using multi-stage sampling approach, which made the findings reliable and representative. Second, compared with other short-term post-disaster studies [28],[29], this long-term post-disaster study had a larger sample size. Therefore, we believe our findings are robust and could provide reference for medical decision-making. Last but not least, our research, for the first time, explored the long-term physical diseases, mental illness, and quality of life of earthquake survivors, and revealed their relationships to some extent.

## References

[1] 95% of reconstruction work has been finished in Wenchuan. Press conference. Available: http://english.cntv.cn/program/newshour/20110510/106972.shtml. Accessed Janurary 30, 2012.

[2] Restoration and Reconstruction in Sichuan Earthquake-hit Areas: a Household Survey Report. Available: http://www.casted.org.cn/upload/news/Attach-20091130163058.pdf. Accessed Janurary 30, 2012.

[3] Zhilan Xiao, Jin Wen, Zhao Hong, Qiuwen Tan, Xiao Lv, et al (2011) A bibliometric analysis on post-earthquake health issue related literature. Chinese Journal of Evidence Based Medicine 11 5 483–488.

[4] Ehring T, Razik S, Emmelkamp PM (2011) Prevalence and predictors of posttraumatic stress disorder, anxiety, depression, and burnout in Pakistani earthquake recovery workers. Psychiatry Res 185 1–2 161–166. 20537401 10.1016/j.psychres.2009.10.018

[5] Fan F, Zhang Y, Yang Y, Mo L, Liu X (2011) Symptoms of posttraumatic stress disorder, depression, and anxiety among adolescents following the 2008 Wenchuan earthquake in China. J Trauma Stress 24 1 44–53. 21351164 10.1002/jts.20599

[6] Ren ZJ, Deng H, Hsu LK (2011) PTSD in a one year old girl after the Wenchuan earthquake in Sichuan, China. Psychiatry 74 1 87–92. 21463173 10.1521/psyc.2011.74.1.87

[7] Salcioglu E, Basoglu M, Livanou M (2007) Post-traumatic stress disorder and comorbid depression among survivors of the 1999 earthquake in Turkey. Disasters 31 2 115–129. 17461919 10.1111/j.1467-7717.2007.01000.x

[8] Varela E, Koustouki V, Davos CH, Eleni K (2008) Psychological consequences among adults following the 1999 earthquake in Athens, Greece. Disasters 32 2 280–291. 18380855 10.1111/j.1467-7717.2008.01039.x

[9] Wang X, Gao L, Zhang H, Zhao C, Shen Y, et al (2000) Post-earthquake quality of life and psychological well-being: longitudinal evaluation in a rural community sample in northern China. Psychiatry Clin Neurosci 54 4 427–433. 10997859 10.1046/j.1440-1819.2000.00732.x

[10] Chou FH, Chou P, Su TT, Ou-Yang WC, Chien IC, et al (2004) Quality of life and related risk factors in a Taiwanese Village population 21 months after an earthquake. Aust N Z J Psychiatry 38 5 358–364. 15144515 10.1080/j.1440-1614.2004.01364.x

[11] Blanchard EB, Jones Alexander J, Buckley TC, Forneris CA (1996) Psychometric properties of the PTSD Checklist (PCL). Behav Res Ther 34 8 669–673. 8870294 10.1016/0005-7967(96)00033-2

[12] Diagnostic and Statistical Manual of Mental Disorders DSM-IV-TR Fourth edition (2000). Washington, DC: American Psychiatric Association.

[13] Dobie DJ, Kivlahan DR, Maynard C, Bush KR, McFall M, et al (2002) Screening for post-traumatic stress disorder in female Veteran's Affairs patients: validation of the PTSD checklist. Gen Hosp Psychiatry 24 6 367–374. 12490337 10.1016/s0163-8343(02)00207-4

[14] Terhakopian A, Sinaii N, Engel CC, Schnurr PP, Hoge CW (2008) Estimating population prevalence of posttraumatic stress disorder: an example using the PTSD checklist. J Trauma Stress 21 3 290–300. 18553416 10.1002/jts.20341

[15] Lang AJ, Laffaye C, Satz LE, Dresselhaus TR, Stein MB (2003) Sensitivity and specificity of the PTSD checklist in detecting PTSD in female veterans in primary care. J Trauma Stress 16 3 257–264. 12816338 10.1023/A:1023796007788

[16] Ware J Jr, Kosinski M, Keller SD (1996) A 12-Item Short-Form Health Survey: construction of scales and preliminary tests of reliability and validity. Med Care 34 3 220–233. 8628042 10.1097/00005650-199603000-00003

[17] Ware JE, Kosinski M, Turner Bowker DM, Gandek B (2005) How to score version 2 of the SF-12 health survey. Boston: Lincoln, R.I. : QualityMetric Inc.

[18] Kun P, Chen X, Han S, Gong X, Chen M, et al (2009) Prevalence of post-traumatic stress disorder in Sichuan Province, China after the 2008 Wenchuan earthquake. Public Health 123 11 703–707. 19892379 10.1016/j.puhe.2009.09.017

[19] Xiang-lan Wang, Jiong Tao, Shenglin Wen (2008) Mental health status of victims of Wenchuan earthquake and affecting factors. Journal of SUN Yat-sen University (Med Sci) 29 4 367–371.

[20] Peng Kun, Xunchui Chen, Manli Chen (2010) Empirical Analysis of Post-disaster Medical Assistance Package Making Based on the Wenchuan Earthquake. Chinese Health Economics 29 11 33–35.

[21] van Griensven F, Chakkraband ML, Thienkrua W, Pengjuntr W, Lopes Cardozo B, et al (2006) Mental health problems among adults in tsunami-affected areas in southern Thailand. JAMA 296 5 537–548. 16882960 10.1001/jama.296.5.537

[22] Jia Z, Tian W, Liu W, Cao Y, Yan J, et al (2010) Are the elderly more vulnerable to psychological impact of natural disaster? A population-based survey of adult survivors of the 2008 Sichuan earthquake. BMC Public Health 10: 172. 20353554 10.1186/1471-2458-10-172 PMC2867995

[23] Zhang Z, Shi Z, Wang L, Liu M (2011) One year later: Mental health problems among survivors in hard-hit areas of the Wenchuan earthquake. Public Health 125 5 293–300. 21524773 10.1016/j.puhe.2010.12.008

[24] Bland SH, O'Leary ES, Farinaro E, Jossa F, Trevisan M (1996) Long-term psychological effects of natural disasters. Psychosom Med 58 1 18–24. 8677284 10.1097/00006842-199601000-00004

[25] Nunnally JC (1978) Psychometric Theory: New York, NY: McGraw-Hill.

[26] Luo X, Lynn George M, Kakouras I, Edwards CL, Pietrobon R, et al (2003) Reliability, validity, and responsiveness of the short form 12-item survey (SF-12) in patients with back pain. Spine (Phila Pa 1976) 28 15 1739–1745. 12897502 10.1097/01.BRS.0000083169.58671.96

[27] Ke X, Liu C, Li N (2010) Social support and Quality of Life: a cross-sectional study on survivors eight months after the 2008 Wenchuan earthquake. BMC Public Health 10: 573. 20863410 10.1186/1471-2458-10-573 PMC2955008

[28] Zhang Y, Ho SM (2011) Risk factors of posttraumatic stress disorder among survivors after the 512 Wenchuan earthquake in China. PLoS One 6 7 223–271. 10.1371/journal.pone.0022371 PMC3143136 21799838

[29] Xu J, Song X (2011) Posttraumatic stress disorder among survivors of the Wenchuan earthquake 1 year after: prevalence and risk factors. Compr Psychiatry 52 4 431–437. 21683180 10.1016/j.comppsych.2010.08.002

